# Evaluation of an Automated Cartridge-Based PCR Assay for the Detection of *Leishmania* spp. DNA in Canine Lymph Node Samples

**DOI:** 10.3390/pathogens15080794

**Published:** 2026-07-27

**Authors:** Eva Spada, Francesca Di Gaudio, Germano Castelli, Federica Bruno, Roberta Perego, Luciana Baggiani, Vito Biondi, Fabrizio Vitale, Michela Tognoni, Daniela Proverbio

**Affiliations:** 1 Dipartimento di Medicina Veterinaria e Scienze Animali (DIVAS), Università degli Studi di Milano, 26900 Lodi, Italy; eva.spada@unimi.it (E.S.); roberta.perego@unimi.it (R.P.); luciana.baggiani@unimi.it (L.B.); daniela.proverbio@unimi.it (D.P.); 2 Centro di Referenza Nazionale per le Leishmaniosi (C.Re.Na.L.), Istituto Zooprofilattico Sperimentale (IZS) della Sicilia A. Mirri, 90129 Palermo, Italy; federica.bruno@izssicilia.it (F.B.); fabrizio.vitale@izssicilia.it (F.V.); 3 Dipartimento di Scienze Veterinarie, Università di Messina, 98168 Messina, Italy; vito.biondi@unime.it; 4 Associazione U.N.A. OdV, Canile Monte Contessa, 16153 Genova, Italy

**Keywords:** canine leishmaniosis, *Leishmania infantum*, point-of-care PCR, lymph node aspirate, molecular diagnostics, veterinary diagnostics

## Abstract

An automated cartridge-based Vcheck M Canine Vector 8 Panel for qualitative detection of *Leishmania* spp. DNA in canine lymph node aspirates, an off-label specimen type, using laboratory qPCR as the comparator, was evaluated. Fifty-seven residual lymph node aspirate suspensions from dogs investigated for suspected canine leishmaniosis (CanL) were tested. Reference qPCR detected *L. infantum* DNA in 29 samples. Vcheck M was positive in 22/29 qPCR-positive samples and negative in 28/28 qPCR-negative samples, corresponding to positive percent agreement/sensitivity of 75.9% (95% CI, 56.5–89.7) and negative percent agreement/specificity of 100.0% (95% CI, 87.7–100.0). Agreement was substantial (Cohen’s kappa, 0.76), and discordance was asymmetric (McNemar *p* = 0.016). Vcheck-negative/qPCR-positive results were mainly observed at low qPCR parasite loads: 6/7 discordant samples contained ≤30 parasites/mL, whereas all samples with ≥500 parasites/mL were Vcheck positive. Among Vcheck-positive clinical samples, Vcheck Ct correlated inversely with log10 qPCR parasite load (Spearman rho = −0.77; *p* < 0.001). In a single-run dilution series, the lowest instrument-positive *L. infantum* standard was 10^3^ parasites/mL. Purified *L. major*, *L. braziliensis*, and *L. tropica* DNA were also detected. Vcheck M showed high specificity as a rapid rule-in test for *Leishmania* spp. detection in canine lymph node aspirates. However, negative results should not exclude infection in symptomatic or strongly suspected dogs and should be confirmed by qPCR, particularly when low parasite burden is plausible or when the result is critical for diagnostic or therapeutic decision-making.

## 1. Introduction

Canine leishmaniosis (CanL), principally caused by *Leishmania infantum* Nicolle, 1908 in the Mediterranean basin, is a chronic vector-borne zoonosis with variable clinical expression, ranging from subclinical infection to severe systemic disease [1,2]. Dogs are a major domestic reservoir for human infection, and diagnosis has individual animal, kennel-management, and public-health implications. Because compatible clinical signs and clinicopathological abnormalities are nonspecific, diagnostic interpretation requires integration of clinical findings with serological, cytological, parasitological, and molecular data [1,2].

PCR assays are widely used for the direct demonstration of *Leishmania* DNA. Lymph node aspirates are easy to obtain and have historically higher sensitivity than blood for molecular detection in dogs and are frequently selected when the aim is to identify tissue infection [3,4]. Real-time PCR assays targeting kinetoplast minicircle DNA (kDNA) are among the most sensitive molecular approaches currently available for the diagnosis of leishmaniasis because the target is present in thousands of copies per parasite. Nevertheless, considerable heterogeneity remains among published assays concerning target selection, analytical sensitivity, specimen type, and quantitative reporting. No universally accepted molecular gold standard currently exists, while there is a growing need for easy-to-use, accessible bench-top molecular assays that can support diagnosis in clinical settings [5]. This emphasizes the importance of independent evaluations of new diagnostic platforms using clinically relevant sample types. Quantitative real-time PCR (qPCR) further provides parasite-load information, which can support diagnosis and monitoring but generally requires dedicated instrumentation, trained staff, and specimen transport to a reference laboratory [6].

In addition to kinetoplast minicircle DNA (kDNA), other molecular targets have been used for detection, quantification, and species identification of *Leishmania*, including the internal transcribed spacer 1 (ITS-1) region, heat-shock protein 70 (hsp70), miniexon, small-subunit rRNA, and other genomic regions [5,7,8]. These targets have been applied through several PCR-based approaches, such as conventional PCR, nested PCR, PCR-restriction fragment length polymorphism (PCR-RFLP), and quantitative real-time PCR, depending on whether the diagnostic aim is detection, parasite-load estimation, or species characterization [5,7,8,9].

Molecular detection of *Leishmania* DNA can be performed on several clinical matrices, including lymph node and bone marrow aspirates, skin, whole blood or buffy coat, and less invasive specimens such as conjunctival or oral swabs. However, diagnostic performance varies markedly according to tissue tropism, parasite burden, sampling method, and analytical target [3,4,10,11].

Automated cartridge-based molecular platforms could reduce turnaround time and broaden access to molecular diagnostics. A portable lab-on-chip qPCR platform has recently been evaluated for *L. infantum* detection in dog samples, supporting the relevance of near-patient molecular approaches for canine leishmaniosis diagnostics [10]. The Vcheck M Canine Vector 8 Panel (SD Biosensor, Gyeonggi-do, Republic of Korea) is a multiplex real-time PCR cartridge intended for use with the Vcheck M10 analyzer, designed for the qualitative detection of target DNA of 8 canine vector-borne pathogen groups in anticoagulated whole blood, including *Leishmania* spp. The manufacturer-stated *Leishmania* targets include *L. infantum*, *L. donovani*, and *L. mexicana*; results are interpreted automatically, while cycle threshold (Ct) values and amplification curves can be reviewed.

Since peripheral blood is generally regarded as a suboptimal matrix for *Leishmania* DNA detection, mainly because of its limited diagnostic sensitivity, the objective of this study was to evaluate the performance of Vcheck M, originally designed for use with whole-blood samples, for the detection of *Leishmania* spp. DNA in canine lymph node aspirates, using an established laboratory qPCR assay as the reference method. Because lymph node aspirate is not the manufacturer-stated specimen type yet represents a key clinically relevant matrix for the diagnosis of CanL, this study should be considered an off-label evaluation of diagnostic performance and analytical feasibility rather than as a verification of the intended-use claim.

## 2. Materials and Methods

### 2.1. Study Design and Samples

This was a prospective diagnostic-accuracy and analytical-feasibility study using residual canine lymph node aspirate samples collected during routine diagnostic investigations. The study was designed and reported in accordance with the Standards for Reporting Diagnostic Accuracy Studies (STARD) principles [12]. From September 2024 onward, one lymph node aspirate per dog suspected for CanL was eligible for inclusion. Suspected CanL was defined by compatible clinical signs, IFAT seropositivity (>1:80), and origin from or residence in an *L. infantum*-endemic area. Lymph node aspirate material was aliquoted for testing with the reference qPCR for diagnostic purposes and with the index assay, Vcheck M Canine Vector 8 Panel (SD Biosensor, Gyeonggi-do, Republic of Korea). The reference qPCR and the index test were performed in different laboratories, with operators blinded to the results of the other assay. After collection, the lymph node aspirates were immediately diluted in 400 μL of phosphate-buffered saline (PBS) and divided into two 200 μL aliquots, stored frozen until analysis. The reference qPCR was performed at the National Reference Center for Leishmaniosis (Centro di Referenza Nazionale per le Leishmaniosi–C.Re.Na.L.), Istituto Zooprofilattico Sperimentale della Sicilia “A. Mirri”, Palermo, Italy. The index test was performed at the Veterinary Transfusion Research Laboratory (REVLab), Department of Veterinary Medicine and Animal Sciences (DIVAS), University of Milan, Lodi, Italy.

No formal a priori sample-size calculation was performed; the sample size was determined by the availability of residual diagnostic lymph node aspirate suspensions that met the inclusion criteria during the study period. Accordingly, the study should be interpreted as a preliminary diagnostic-performance and analytical-feasibility evaluation.

The use of residual diagnostic samples involved no additional invasive procedures performed solely for research purposes. According to the University of Milan regulations and the Ethical Committee decision of 29 October 2012, renewed with protocol no. 02-2016, formal approval was not required for the use of residual samples collected during routine veterinary care with owner consent.

### 2.2. Index Test

The Vcheck M Canine Vector 8 Panel cartridge is a disposable plastic device intended for whole-blood samples that contains all reagents required for a fully automated molecular assay. Each cartridge was run on the Vcheck M10 system (SD Biosensor, Suwon, Republic of Korea) according to the manufacturer’s established workflow, with adaptation for lymph node aspirate suspensions. Briefly, 100 μL of PBS aspirate suspension was mixed with 500 μL of kit buffer, and 600 μL of the resulting mixture was loaded into the cartridge. The instrument performs automated nucleic acid extraction, amplification, detection, and result interpretation in 70 min for each analyzed sample. Although only the *Leishmania* spp. result was considered for this study, Vcheck M Canine Vector 8 Panel is a multiplex assay targeting eight pathogen groups: *Ehrlichia* spp., *Hepatozoon* spp., canine hemotropic mycoplasmas, *Anaplasma* spp., *Rickettsia rickettsii*, *Babesia* spp., *Leishmania* spp., and *Bartonella* spp. For this analysis, the prespecified index-test result was the automated qualitative *Leishmania* output (positive/negative). Ct values were extracted and annotated. The limit of detection (LOD) on a blood sample claimed by the manufacturer is 1.2 × 10^4^ copies/mL of *Leishmania* spp.

The exact primer/probe sequences and molecular target of the Vcheck M Leishmania channel were not disclosed by the manufacturer and are therefore considered proprietary; this limitation was considered when interpreting the comparison with the reference qPCR assay.

### 2.3. Reference qPCR

Reference test for *L. infantum* was performed by IZS using a qPCR assay described by Castelli et al. [6]. DNA extraction was performed using the PureLink Genomic DNA Mini Kit (Thermo Fisher Scientific, Waltham, MA, USA). qPCR was performed on a QuantStudio 3 instrument (Life Technologies, Waltham, MA, USA) in 20-μL reactions containing 10 μL SsoAdvanced Universal Probes Supermix (Bio-Rad, Hercules, CA, USA), 0.25 μM QLeish probe, 0.3 μM of each primer, and 2 μL extracted DNA at 10 ng/μL. A 10-fold serial dilution of *L. infantum* parasite DNA corresponding to 10^6^ to 1 parasite/mL was used as the standard curve. Cycling consisted of 95 °C for 10 min, followed by 40 cycles of 95 °C for 15 s and 60 °C for 35 s. As reported in Castelli et al. [6], the molecular target used for qPCR detection is the minicircle DNA of the *L. infantum* kinetoplast (kDNA). DNA concentration and purity were assessed using a NanoDrop spectrophotometer (Thermo Fisher Scientific, Waltham, MA, USA). DNA concentration was determined by measuring absorbance at 260 nm, while purity was evaluated using the A260/A280 and A260/A230 absorbance ratios.

The reference qPCR result was considered positive when the estimated parasite load was ≥1 parasite/mL, according to the assay criteria previously described [6]. In the original assay validation, the standard curve showed an R^2^ of 0.99 and a mean slope of −3.13, corresponding to high amplification efficiency. The lower detection threshold used for positivity was 1 parasite/mL. Reference parasite loads were reported as parasites/mL as generated by the reference laboratory.

### 2.4. Analytical Sensitivity and Ability to Identify Species Across Leishmania spp.

To estimate analytical sensitivity, a concentrated *Leishmania (Leishmania) infantum* (MCAN/IT/2025/1265) standard (10^8^ parasites/mL) supplied by C.Re.Na.L. was serially diluted 10-fold in PBS and tested once per dilution. To assess the ability to identify *Leishmania* species different from *L. infantum*, *L. donovani* and *L. mexicana*, three samples with different *Leishmania* spp. i.e., *L. (L) major* (MHOM/SU/1973/5ASKH), *L. (L) tropica* (MHOM/SU/1974/K27), and *L. (Viannia) braziliensis* (MHOM/BR/1975/M2904) were tested.

### 2.5. Statistical Analysis

Sensitivity, specificity, positive predictive value, negative predictive value, and accuracy were calculated with 95% confidence intervals. Cohen’s kappa quantified agreement beyond chance. Exact McNemar testing compared discordant paired qualitative results. Sensitivity was also summarized after excluding qPCR-positive samples. The association between Vcheck M Ct and log10 reference qPCR load was evaluated by linear regression and Spearman correlation among Vcheck-positive samples. Parasite loads are reported as parasites/mL; log10-transformed values were used to improve data visualization and reduce skewness. Analyses were performed with Medcalc software (MedCalc Statistical Software version 23.2.1, MedCalc Software Ltd., Ostend, Belgium).

## 3. Results

### 3.1. Samples

The analyzed samples were 57 canine lymph node aspirates from 57 different dogs tested between September 2024 and November 2025. Specimens originated from three sources: 8 samples were from privately owned dogs evaluated at the University Veterinary Teaching Hospital of the University of Milan, in northern Italy, 10 from dogs in a private Genoa canine shelter, in northern Italy, and 39 samples from privately owned and shelter dogs from Sicily, in southern Italy.

For spatial characterization, the three sample-source locations were reported at center level as follows: University Veterinary Teaching Hospital, University of Milan, Lodi, Lombardy (approximately 45.31° N, 9.50° E); Canile Monte Contessa, Genoa, Liguria (approximately 44.43° N, 8.84° E); and Istituto Zooprofilattico Sperimentale della Sicilia A. Mirri, Palermo, Sicily (approximately 38.11° N, 13.35° E).

### 3.2. Agreement with Reference qPCR

Reference qPCR detected *L. infantum* DNA in 29 specimens and was negative in 28 specimens. Vcheck M was positive in 22 of 29 qPCR-positive specimens and negative in all 28 qPCR-negative specimens (Table 1). Therefore, true-positive samples were 22, false-negative samples were 7, true-negative samples were 28, and false-positive samples were 0. This corresponded to sensitivity of 75.9% (95% CI, 56.5–89.7), specificity of 100.0% (95% CI, 87.7–100.0), positive predictive value of 100.0% (95% CI, 84.6–100.0), negative predictive value of 80.0% (95% CI, 63.1–91.6), and accuracy of 87.7% (95% CI, 76.3–94.9). Cohen’s kappa was 0.76, and the exact McNemar *p* value was 0.016.

### 3.3. Effect of Parasite Load

Seven specimens were Vcheck M negative but qPCR positive. Six had low qPCR loads (1.44, 2.12, 2.20, 8.04, 11, and 30 parasites/mL), while one specimen with 420 parasites/mL was negative by Vcheck M. When sensitivity was recalculated after excluding low-load qPCR-positive samples, Vcheck M performance improved progressively with increasing parasite burden. Sensitivity was 84.6% for samples with ≥5 parasites/mL, 88.0% for samples with ≥10 parasites/mL, 95.5% for samples with ≥40 parasites/mL, and 100% for samples with ≥500 parasites/mL (Table 2). Specificity remained 100% in all strata, since no false-positive Vcheck M result for *Leishmania* spp. was detected.

### 3.4. Ct-Load Association

**Figure 1:**
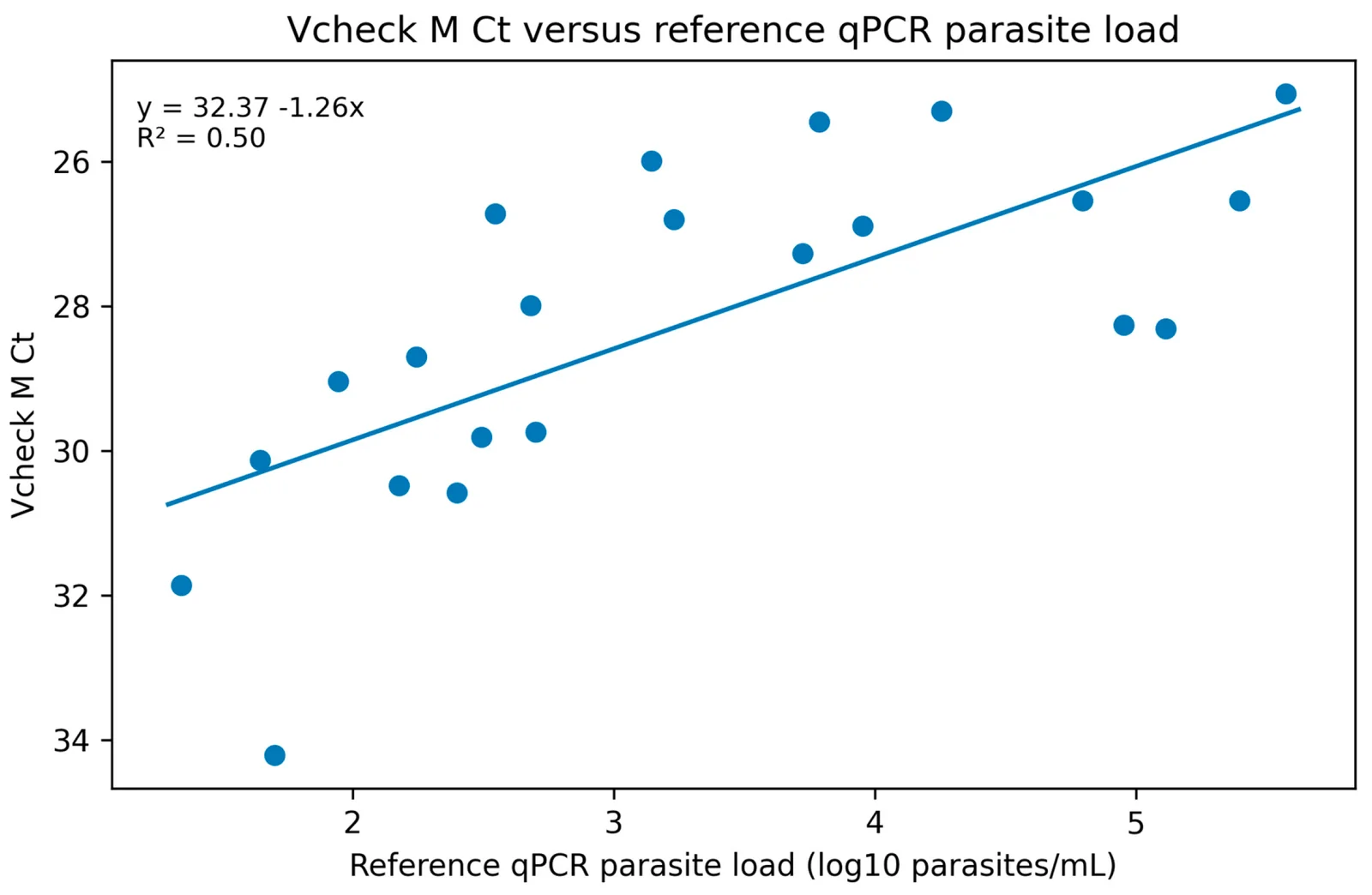
Vcheck M Leishmania Ct values versus reference qPCR parasite load in Vcheck-positive lymph node aspirate specimens (n = 22). Parasite load is shown as log10 parasites/mL. The y-axis is inverted so lower Ct values appear higher on the plot. The regression equation was Vcheck M Ct = 32.37 − 1.26 × log10(qPCR parasite load), with R^2^ = 0.50.

Among the 22 Vcheck-positive qPCR-positive specimens with reported Ct values, Vcheck M Ct was inversely associated with log10 reference qPCR load. The linear-regression equation was Vcheck M Ct = 32.37 − 1.26 × log10(qPCR parasite load), with R^2^ = 0.50 (r = −0.71, *p* = 0.0002); Spearman rho was −0.77 (*p* < 0.001) (Figure 1). The association was statistically significant but not strong enough to use Vcheck M Ct as an interchangeable quantitative parasite-load measure.

### 3.5. Analytical Sensitivity and Ability to Identify Across Leishmania Species

In a single 10-fold serial dilution of the *L. infantum* standard (n = 1 per concentration), Vcheck M dilutions from 10^7^ to 10^3^ parasites/mL were positive, with Ct values from 18.86 to 32.06. The 10^2^ parasites/mL dilution showed late amplification (Ct 35.39) but was interpreted as negative by the instrument (Table 3). Because each dilution was tested once, mean Ct values and standard deviations could not be calculated. DNA from *L. major* (Ct 18.83), *L. braziliensis* (Ct 19.95), and *L. tropica* (Ct 17.01) was detected by the *Leishmania* channel and interpreted as positive.

## 4. Discussion

This study provides an independent, off-label evaluation of an automated veterinary cartridge PCR system for detecting *Leishmania* DNA in canine lymph node aspirate. The main finding is a high specificity signal: no qPCR-negative lymph node specimen produced a Vcheck M *Leishmania* positive result. This suggests that a positive result, when obtained from a properly collected and processed lymph node aspirate, is strongly supportive of *Leishmania* DNA detection in the tested specimen and should be interpreted together with clinical and epidemiological data.

However, sensitivity was strongly influenced by parasite burden. The burden-dependent sensitivity is clinically important. Most discordant qPCR-positive/Vcheck-negative specimens had reference loads below 30 parasites/mL, and all clinical specimens at or above 500 parasites/mL were detected. This pattern is consistent with the expected challenge of detecting low-copy targets in small input volumes and with the prior literature emphasizing that molecular results depend on specimen type, parasite distribution, and target burden [3,4,6]. The clustering of Vcheck M false-negative results among specimens with very low parasite loads in the present study supports the concept that analytical sensitivity becomes a critical determinant when parasite DNA approaches the lower limits of detection of the assay. These findings are consistent with previous observations that molecular detection of *L. infantum* is strongly influenced by parasite burden. Francino et al. reported that conventional PCR failed to detect samples containing fewer than approximately 30 parasites/mL, whereas quantitative real-time PCR remained positive across a substantially wider dynamic range. Their work highlighted that parasite quantification provides clinically relevant information beyond simple positive/negative classification and may improve the interpretation of low-level infections and treatment monitoring [13].

The assay should therefore not be positioned as a replacement for reference qPCR in all use cases. In line with the manufacturer’s interpretation criteria, negative Vcheck M results do not preclude *Leishmania* spp. infection diagnosis and should not be used as the sole basis for treatment or other patient-management decisions. Negative results should be interpreted together with the animal’s history, clinical and clinicopathological findings, serological status, and epidemiological context. When a negative result is not consistent with the overall clinical picture, particularly in dogs with strong clinical suspicion, previous or ongoing treatment, or when low-level parasite DNA is expected, confirmation by a more sensitive reference laboratory qPCR is warranted. Reference qPCR also remains preferable for quantitative monitoring and for diagnostic questions requiring detection of very low parasite burdens. Conversely, in dogs with compatible clinical signs, positive serology, and moderate-to-high tissue parasite burden, a positive Vcheck M result may provide a rapid and reliable rule-in indication for *Leishmania* spp. infection.

The rapid turnaround time has practical clinical implications. A same-day molecular result may support earlier diagnostic orientation, reduce delays associated with shipment to external laboratories, and facilitate adoption of molecular testing in veterinary clinics or shelters that do not have specialized molecular infrastructure. In this setting, the platform is best positioned as a rapid rule-in tool when a positive result is obtained, while negative results in high-suspicion dogs require confirmatory testing.

The observed performance should also be interpreted in the context of cartridge-based molecular diagnostics. Fully automated systems generally reduce manual extraction, hands-on time, contamination risk, operator dependence, and turnaround time, but this simplification may be associated with a lower ability to detect very small amounts of target DNA compared with optimized reference laboratory qPCR workflows. Therefore, the moderate sensitivity observed in low-load samples was not unexpected and reflects the intended near-patient role of this technology rather than equivalence to a highly sensitive quantitative reference assay.

This research adds useful but preliminary information on the use of a bench-top PCR for canine lymph node aspirate evaluation for *Leishmania* spp. infection. The *L. infantum* standard dilution series analyses suggested reliable instrument positivity through 10^3^ parasites/mL, while 10^2^ parasites/mL produced a late Ct but a negative qualitative result. This distinction underscores that the manufacturer’s algorithm, not Ct alone, determines the reported qualitative result. The detection of *L. major*, *L. braziliensis*, and *L. tropica* indicates broader *Leishmania* DNA recognition than the species list emphasized in the package insert, but these data were generated with purified DNA and do not establish clinical performance for those species. Broader detection of non-*L. infantum* species is biologically plausible because many molecular assays targeting kinetoplast minicircle DNA amplify conserved regions shared across multiple *Leishmania* species. Dantas-Torres et al. demonstrated cross-detection of *L. braziliensis* using primers originally developed for *L. infantum*, highlighting the broad analytical reactivity that may result from the high conservation of selected kDNA regions [14]. Therefore, the positive results obtained with *L. major*, *L. braziliensis*, and *L. tropica* DNA in the present study are consistent with previous observations and suggest that the Vcheck M Leishmania channel may recognize a wider range of *Leishmania* species than those explicitly listed by the manufacturer.

The choice of lymph node aspirates was based on their recognized diagnostic relevance in CanL [3,4]. Although this matrix is outside the manufacturer-stated whole-blood indication, lymphoid tissue is one of the main sites of parasite persistence and is frequently associated with higher parasite loads than peripheral blood in infected dogs. For this reason, lymph node aspirate is commonly used for cytological, parasitological, and molecular diagnosis and represents a clinically meaningful matrix for evaluating whether a rapid molecular platform can detect tissue-associated infection. Although lymph node aspirate is not the manufacturer-stated specimen type, this study has several strengths. First, it evaluates a bench-top molecular platform that could facilitate a rapid and targeted diagnostic approach to CanL by enabling *Leishmania* spp. DNA detection in a clinically relevant tissue matrix. Second, the study used field-collected lymph node aspirates, an established quantitative qPCR assay as the reference method, and explicitly assessed how parasite burden affected diagnostic performance.

Limitations include the modest sample size and the use of a specimen type outside the manufacturer-stated whole-blood indication. Moreover, predictive values should be interpreted within the high-suspicion population evaluated in this study and may differ in screening settings or in populations with lower disease prevalence. In addition, direct quantitative comparisons between molecular platforms should be interpreted cautiously because parasite-load estimates can be influenced by target copy number, extraction efficiency, amplification chemistry, and calibration strategy. Variability among qPCR methodologies has been recognized as a major challenge in the standardization of molecular diagnosis for leishmaniasis [5]. Additional limitations are the absence of a formal a priori power calculation, the convenience nature of the residual diagnostic sample set, the lack of paired blood samples from the same dogs, and the unavailability of the proprietary Vcheck M primer/probe sequences and molecular target. These aspects limit direct comparison with the manufacturer-stated whole-blood intended-use claim and should be considered when extrapolating the results to other clinical contexts.

Implementation costs and cost-effectiveness were not assessed in the present study. Because instrument and cartridge prices may vary according to country, distributor, procurement conditions, and institutional agreements, formal economic evaluation should be addressed separately, particularly for use in resource-limited endemic settings.

Future studies should include larger and more diverse populations, asymptomatic infected dogs, and paired comparisons among blood, lymph node, bone marrow, skin, and conjunctival or oral swab specimens. Longitudinal studies during treatment would also clarify whether Vcheck M can support clinical follow-up, although reference qPCR remains preferable for quantitative monitoring. Validation in different endemic regions and with additional *Leishmania* species is also warranted.

## 5. Conclusions

In conclusion, this study provides an independent, off-label evaluation of Vcheck M applied to canine lymph node aspirates, a clinically relevant matrix for the diagnosis of CanL but not the manufacturer-stated specimen type. The platform showed excellent observed specificity but parasite-load-dependent sensitivity for *Leishmania* DNA detection. It may be useful as a rapid point-of-care or near-patient rule-in molecular tool, particularly in symptomatic dogs with moderate-to-high tissue parasite burden. However, negative results require cautious interpretation and should be followed by reference qPCR when clinical suspicion, previous treatment, or monitoring needs remain.

## Figures and Tables

**Table 1:** Diagnostic performance of Vcheck M for *Leishmania* spp. DNA detection in the lymph node aspirate samples.

Metric	Numerator/Denominator	Estimate (%)	95% CI
Sensitivity	22/29	75.9	56.5–89.7
Specificity	28/28	100.0	87.7–100.0
Positive predictive value	22/22	100.0	84.6–100.0
Negative predictive value	28/35	80.0	63.1–91.6
Accuracy	50/57	87.7	76.3–94.9

95% CI: 95% Confidence interval.

**Table 2:** Diagnostic performance of Vcheck M for *Leishmania* spp. DNA detection in the lymph node aspirate samples: burden-stratified sensitivity in the set of analyzed samples.

Included qPCR-Positive Samples	True-Positive	False-Negative	Sensitivity, %	95% CI
All qPCR-positive samples	22	7	75.9	56.5–89.7
≥5 parasites/mL	22	4	84.6	65.1–95.6
≥10 parasites/mL	22	3	88.0	68.8–97.5
≥40 parasites/mL	21	1	95.5	77.2–99.9
≥100 parasites/mL	18	1	94.7	74.0–99.9
≥500 parasites/mL	12	0	100.0	73.5–100

qPCR quantitavive real-time PCR; 95% CI: 95% Confidence interval.

**Table 3:** Descriptive analytical sensitivity of Vcheck M for *Leishmania* spp. DNA detection in a single-run 10-fold dilution series of an *L. infantum* standard.

Material	Concentration	log10 Concentration	n	Detection Rate	Qualitative Result	Ct
*L. infantum* standard	10^7^ parasites/mL	7	1	1/1 (100%)	Positive	18.86
*L. infantum* standard	10^6^ parasites/mL	6	1	1/1 (100%)	Positive	21.93
*L. infantum* standard	10^5^ parasites/mL	5	1	1/1 (100%)	Positive	25.39
*L. infantum* standard	10^4^ parasites/mL	4	1	1/1 (100%)	Positive	29.24
*L. infantum* standard	10^3^ parasites/mL	3	1	1/1 (100%)	Positive	32.06
*L. infantum* standard	10^2^ parasites/mL	2	1	0/1 (0%)	Negative	35.39

Ct: cycle threshold.

## Data Availability

The data presented in this study are available from the corresponding authors upon reasonable request, subject to institutional and privacy restrictions.

## Abbreviations

The following abbreviations are used in this manuscript:

CanL	Canine Leishmaniosis
STARD	Standards for Reporting Diagnostic Accuracy Studies
IFAT	Indirect fluorescent antibody test
qPCR	Real-time polymerase chain reaction
Ct	Cycle threshold
kDNA	kinetoplast minicircle DNA
LOD	Limit of detection

## References

[1] Solano-Gallego L., Mirá G., Koutinas A., Cardoso L., Pennisi M.G., Ferrer L., Bourdeau P., Oliva G., Baneth G. (2011). LeishVet guidelines for the practical management of canine leishmaniosis. Parasit. Vectors.

[2] Solano-Gallego L., Koutinas A., Miró G., Cardoso L., Pennisi M.G., Ferrer L., Bourdeau P., Oliva G., Baneth G. (2009). Directions for the diagnosis, clinical staging, treatment and prevention of canine leishmaniosis. Vet. Parasitol..

[3] Reale S., Maxia L., Vitale F., Glorioso N.S., Caracappa S., Vesco G. (1999). Detection of *Leishmania infantum* in dogs by PCR with lymph node aspirates and blood. J. Clin. Microbiol..

[4] Manna L., Vitale F., Reale S., Caracappa S., Pavone L.M., Morte R.D., Cringoli G., Staiano N., Gravino A.E. (2004). Comparison of different tissue sampling for PCR-based diagnosis and follow-up of canine visceral leishmaniosis. Vet. Parasitol..

[5] Galluzzi L., Ceccarelli M., Diotallevi A., Menotta M., Magnani M. (2018). Real-time PCR applications for diagnosis of leishmaniasis. Parasit. Vectors.

[6] Castelli G., Bruno F., Reale S., Catanzaro S., Valenza V., Vitale F. (2021). Diagnosis of leishmaniasis: Quantification of parasite load by a real-time PCR assay with high sensitivity. Pathogens.

[7] Schönian G., Nasereddin A., Dinse N., Schweynoch C., Schallig H.D.F.H., Presber W., Jaffe C.L. (2003). PCR diagnosis and characterization of *Leishmania* in local and imported clinical samples. Diagn. Microbiol. Infect. Dis..

[8] Montalvo A.M., Fraga J., Monzote L., Montano I., De Doncker S., Dujardin J.C., Van Der Auwera G. (2010). Heat-shock protein 70 PCR-RFLP: A universal simple tool for *Leishmania* species discrimination in the New and Old World. Parasitology.

[9] Fisa R., Riera C., Gállego M., Manubens J., Portús M. (2001). Nested PCR for diagnosis of canine leishmaniosis in peripheral blood, lymph node and bone marrow aspirates. Vet. Parasitol..

[10] Latrofa M.S., Cereda M., Louzada-flores V.N., Dantas-torres F., Otranto D. (2024). Q3 lab-on-chip real-time PCR for the diagnosis of *Leishmania infantum* infection in dogs. J. Clin. Microbiol..

[11] Maia C., Ramada J., Cristóvão J., Gonçalves L., Campino L. (2009). Diagnosis of canine leishmaniasis: Conventional and molecular techniques using different tissues. Vet. J..

[12] Bossuyt P.M., Reitsma J.B., Bruns D.E., Gatsonis C.A., Glasziou P.P., Irwig L., Lijmer J.G., Moher D., Rennie D., De Vet H.C.W. (2015). STARD 2015: An updated list of essential items for reporting diagnostic accuracy studies. BMJ.

[13] Francino O., Altet L., Sánchez-Robert E., Rodriguez A., Solano-Gallego L., Alberola J., Ferrer L., Sánchez A., Roura X. (2006). Advantages of real-time PCR assay for diagnosis and monitoring of canine leishmaniosis. Vet. Parasitol..

[14] Dantas-Torres F., da Silva Sales K.G., Gomes da Silva L., Otranto D., Figueredo L.A. (2017). Leishmania-FAST15: A rapid, sensitive and low-cost real-time PCR assay for the detection of *Leishmania infantum* and *Leishmania braziliensis* kinetoplast DNA in canine blood samples. Mol. Cell. Probes.

